# The Role of Extracellular Vesicles in Bone Regeneration and Associated Bone Diseases

**DOI:** 10.3390/cimb46090548

**Published:** 2024-08-23

**Authors:** Xinyue Wan, Wenjie Zhang, Lingyan Dai, Liang Chen

**Affiliations:** 1 School of Medicine, Chongqing University, Chongqing 400030, China; 202337021023@stu.cqu.edu.cn (X.W.); 202337021070t@stu.cqu.edu.cn (W.Z.); 202337021017@stu.cqu.edu.cn (L.D.); 2 Department of Bone and Soft Tissue Oncology, Chongqing University Cancer Hospital, Chongqing University School of Medicine, Chongqing 400030, China

**Keywords:** EVs, bone regeneration, mechanisms, diagnosis, treatment

## Abstract

Extracellular vesicles (EVs) are nanoscale particles with a lipid bilayer membrane structure secreted by various cell types. Nearly all human cells secrete EVs, primarily mediating intercellular communication. In recent years, scientists have discovered that EVs can carry multiple biological cargos, such as DNA, non-coding RNAs (ncRNAs), proteins, cytokines, and lipids, and mediate intercellular signal transduction. Bone is a connective tissue with a nerve supply and high vascularization. The repair process after injury is highly complex, involving interactions among multiple cell types and biological signaling pathways. Bone regeneration consists of a series of coordinated osteoconductive and osteoinductive biological processes. As mediators of intercellular communication, EVs can promote bone regeneration by regulating osteoblast-mediated bone formation, osteoclast-mediated bone resorption, and other pathways. This review summarizes the biogenesis of EVs and the mechanisms by which EV-mediated intercellular communication promotes bone regeneration. Additionally, we focus on the research progress of EVs in various diseases related to bone regeneration. Finally, based on the above research, we explore the clinical applications of engineered EVs in the diagnosis and treatment of bone regeneration-related diseases.

## 1. Introduction

Bone defects are severe orthopedic conditions caused by trauma, osteoporosis-induced fractures, osteoarthritis, and malignant tumors [1]. With the global aging population and environmental climate changes, the incidence of bone defects has significantly increased [2]. Recent epidemiological data confirms that osteoporosis leads to at least 8.9 million fractures worldwide annually, with over 2 million bone repair surgeries conducted each year in the United States; in the United Kingdom, over 17.8 million people suffer from bone diseases [3]. In mainland China, the prevalence of osteoporosis and vertebral fractures is also increasing yearly [4]. Unfortunately, current bone regeneration research falls far short of meeting clinical demands. In clinical practice, autografting is by far not the most commonly used technique for bone regeneration therapy. However, if grafting is necessary, autologous bone grafting remains the gold standard of treatment. Autologous transplantation also has serious side effects (e.g., infection, inflammation, hematoma, etc.) and limitations. Bone tissue engineering, based on this challenge, continually studies natural grafts and synthetic materials. Improving the biocompatibility and mechanical strength of these biomaterials, reducing immune rejection, and addressing economic issues are current research focuses in the field of bone regeneration. However, with the increasing research on EVs in recent years, it is suggested that EVs may become a new treatment modality for bone regeneration-related diseases.

EVs are nanoparticles secreted by various cells, ranging from 30 nm to 10 μm in diameter. Various bioactive molecules such as DNA, proteins, non-coding RNAs, and cytokines are loaded into EVs, which reach various parts of the body through the circulatory system, mediating intercellular communication. EVs can be broadly categorized into three types based on their diameter, morphology, and structure: exosomes, microvesicles, and apoptotic bodies [5]. Exosomes, discovered in mature erythrocytes in the 1980s, are nanometer-sized vesicles of 30 to 150 nm, enveloped by a bilayer phospholipid membrane. Microvesicles are larger, with diameters of 50–1000 nm and regular shapes, formed by budding from the cell membrane. On the other hand, apoptotic bodies are larger vesicles of 500–2000 nm in size, released by cells during apoptosis and containing cellular debris and particles [6]. In recent years, numerous studies have confirmed that bioactive molecules carried by EVs play critical roles in the development, diagnosis, and treatment of various diseases by regulating cellular signaling pathways. EVs and their bioactive molecules can modulate the functions of osteoblasts, osteoclasts, immune cells, and mesenchymal stromal, playing a significant role in promoting bone regeneration [7]. Due to their low immunogenicity, targeting ability, and biocompatibility, EVs are increasingly used as an alternative to cell transplantation in the field of bone regeneration [8]. Additionally, EVs can easily penetrate blood vessels, promoting the differentiation of osteoblasts and mesenchymal stromal cells, thus having great potential for osteogenesis [9]. The main challenges in using engineered EVs in bone tissue engineering are the difficulty in locating and aggregating EVs near bone tissues and the precise control of EV release without appropriate scaffold support [10].

In this review, we focus on summarizing the specific mechanisms by which EVs promote bone regeneration by regulating relevant cells. We also review the applications and challenges of engineered EVs in the treatment of bone regeneration-related diseases. In the future, engineered EVs will undoubtedly play a critical role in the field of bone regeneration, increasing treatment opportunities and quality of life for patients with bone defects.

## 2. Biogenesis and Sorting of EVs

EVs are classified into exosomes, microvesicles, and apoptotic bodies based on their diameter, origin, and morphology. Among them, apoptotic bodies are secreted by dying cells and are eventually engulfed and degraded by macrophages. Therefore, exosomes and microvesicles are the primary carriers for transporting cargo, with their biogenesis being highly complex and regulated by various cytokines and signaling pathways.

### 2.1. Exosomes

The generation of exosomes is a very intricate and precise process, involving multiple steps and regulatory molecular mechanisms [11]. Initially, primary endocytic vesicles fuse to form early endosomes (EEs). At this stage, EEs carry a large number of biomolecular cargos to form scaffold proteins. Subsequently, EEs selectively undergo two pathways: one carries the biomolecular cargos to the plasma membrane to become “recycling endosomes”, and the other undergoes degradation to form “late endosomes” (LEs), forming intraluminal vesicles (ILVs) through inward budding, encapsulating intracellular proteins, and eventually forming “multivesicular bodies” (MVBs). The formation of ILVs relies on the endosomal sorting complexes required for transport (ESCRT), including ESCRT-0, ESCRT-I, ESCRT-II, and ESCRT-III, which work together to sort specific proteins, DNA, and RNA into ILVs [12]. Notably, the aggregation of ESCRT-III induces endosomal curvature and separation, forming ILVs, also known as MVBs. Proteins such as ALIX and syntenin play important roles in this process by interacting with ESCRT-III to promote ILV formation [13]. Subsequently, ILVs and associated biomolecules are packaged into MVBs, which selectively fuse with the plasma membrane to release ILVs as exosomes or fuse with lysosomes for internal degradation. The fate of MVBs is determined by various regulatory factors, including protein ubiquitination status, ATPase VPS4, and tetraspanin CD63 [14]. Additionally, the cell type and extracellular environmental conditions influence MVB biogenesis and release. Finally, the soluble N-ethylmaleimide-sensitive factor attachment protein receptor (SNARE) complex drives the fusion of MVBs with the plasma membrane, leading to exosome release into the extracellular environment. Various proteins such as VAMP7, YKT6, and SNAP23 are involved in this process [15]. The biogenesis of exosomes is not only an extremely complex biological and genetic process but also crucial for intracellular communication and disease progression.

### 2.2. Microvesicles

The formation of microvesicles (MVs) is a complex process regulated by multiple signaling pathways and cytokines. Unfortunately, our current research in this field is still very limited. Studies over the past 20 years have identified a series of factors that regulate MV release. For example, GTPases (such as the Rho family), ARF, and other cytoskeletal elements play key roles in this regulation [7]. Studies have confirmed that the activation of ARF6 enhances MV production in immune cells, while RhoA and its effectors are involved in MV formation in other cells [16]. Acid sphingomyelinase (ASM) and ATP receptor P2×7 have been reported to participate in MV formation in vascular and stromal cells. ARRDC1-mediated MV formation is highly similar to the membrane shedding induced by viral gag [17]. In contrast to exosome biogenesis, the formation of serum microvesicles (sMVs) involves ARF6 GDP/GTP cycling and RHOA-dependent cytoskeletal rearrangement, directly budding or shedding from the plasma membrane. sMV formation does not require exocytosis and can be released under various stress factors (such as ionizing radiation, DNA strand breaks, and increased intracellular calcium levels) [18]. Additionally, membrane surface receptors such as ALG-2, ARRDC1, and other factors promote MV release through the ESCRT pathway protein complex [19]. The biogenesis of MVs involves a complex regulatory network composed of GTPases, cytoskeletal elements, and intracellular signaling pathways.

### 2.3. Apoptotic Bodies

**Figure 1:**
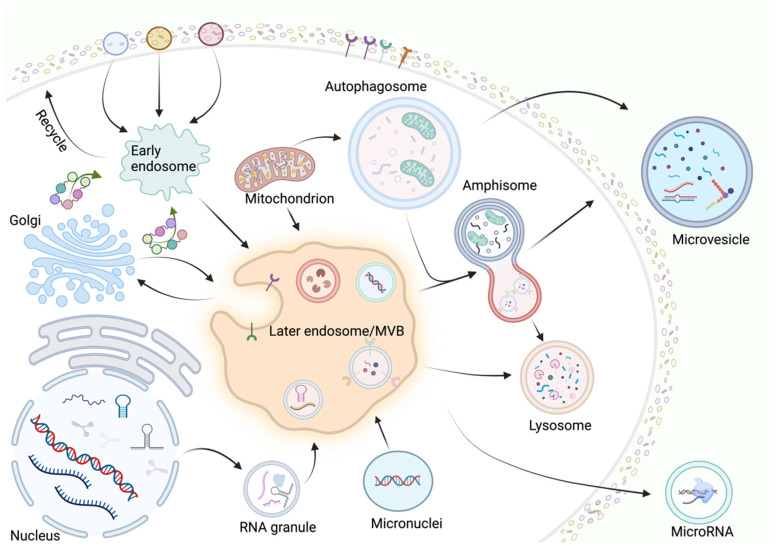
The primary biogenesis mechanisms of EVs involve complex regulatory networks. Extracellular vesicles are categorized as exosomes, microvesicles, and apoptotic vesicles based on their diameter, origin, and shape. The biogenesis of exosomes and microvesicles, as the main carriers for transporting cargos, is very complex and involves the regulation of multiple cytokines and signaling pathways. Primary endosomal vesicles fuse to form early endosomes, followed by EEs carrying a large cargo of biomolecules to form backbone proteins, and then EEs selectively undergo two pathways: one pathway is to become “regenerating endosomes” and the other is to form “late endosomes” (LEs). Microvesicle generation is a complex process in which the release is mainly regulated by factors such as GTPases (e.g., Rho family), ARF, and other cytoskeletal elements. This figure was created by BioRender (https://app.biorender.com (accessed on 30 July 2024)).

The biogenesis of apoptotic bodies (ApoEVs) is strictly regulated by specific pathways. The mechanisms of biogenesis vary among different subtypes of ApoEVs. The formation process of ApoEVs mainly includes three stages: membrane blebbing, membrane protrusion formation, and apoptotic body (ApoBD) generation [20]. Caspases play crucial roles in ApoBD formation. Studies have found that overexpression of Bc-2 can inhibit ApoBD formation by suppressing caspase activity. Caspases significantly promote the membrane blebbing and membrane protrusion formation processes during ApoBD formation [21]. Additionally, ADP-ribose polymer is critical for ApoBD formation in HL-60 cells, and inhibiting mono-ADP-ribosyltransferase activity can also suppress ApoBD generation. Functional microtubules and myosin light chain kinase (MLCK) are important for nuclear contraction and loading nuclear material into membrane vesicles derived from apoptotic cells [22]. The fungal metabolite cytochalasin B inhibits ApoBD formation by preventing actin polymerization, demonstrating the importance of microfilament assembly in ApoBD formation. In Jutkat T cells, inhibiting the plasma membrane channel pannexin 1 (PANX1) or suppressing Rho-associated protein kinase 1 (ROCK1) and PANX1 promotes ApoBD formation, with the former enhancing membrane blebbing during apoptosis and the latter producing beaded apoptotic protrusions [23]. In this section, we have detailed the biogenesis mechanisms of EVs (Figure 1).

## 3. Regulation of Bone Regeneration by EVs

### 3.1. EV-Mediated Intercellular Interactions

Extracellular vesicles (EVs) from various sources play significant roles in bone regeneration by mediating cell-to-cell communication. EVs from different origins, such as mesenchymal stromal cells, osteoblasts, and osteoclasts, promote bone regeneration through various mechanisms. Additionally, EVs provide nutritional support for bone regeneration by promoting angiogenesis. Lastly, EVs facilitate bone regeneration by directly promoting cartilage growth, with multiple mechanisms working together to enhance bone regeneration.

#### 3.1.1. Macrophages (Mφ)

EVs play a crucial biological role in mediating communication between macrophages (Mφ) and other cells through the bioinformational molecules they carry. Known for their involvement in intercellular communication, EVs offer new avenues for understanding bone regeneration and developing new diagnostic and therapeutic options. As dynamic cells, mesenchymal stromal cell-derived macrophages play important roles in the occurrence and treatment of metabolic and inflammation-related diseases. Macrophages, being highly heterogeneous and plastic, can polarize into different types (M0, M1, and M2) in vivo depending on the stimuli and microenvironment [24]. Recent studies have found that Mφ exhibit different phenotypes and functions depending on the tissue, microenvironment, and stimulation, rather than just being categorized into M0, M1, and M2 types. Chu et al. demonstrated that the close interaction between bone marrow mesenchymal stem cells (BMSCs) and Mφ is crucial for maintaining bone homeostasis. In most bone loss diseases (e.g., osteoporosis and osteoarthritis), BMSCs activate Mφ to enhance tissue repair when promoting osteogenic differentiation. Mφ mainly express as the M2 type, regulating osteoblast proliferation, differentiation, survival, and normal function, ultimately maintaining bone homeostasis [25]. Ekstrom et al. showed that EVs derived from LPC-stimulated human MCs could promote osteogenic differentiation by significantly enhancing the secretion of osteogenic markers Runt-related transcription factor 2 (RUNX2) and BMP-2 in hBMSCs [26]. Schwann cell-derived exosomes promote the osteogenesis of BMSCs by inducing M2-type Mφ polarization and upregulating the TGF-β1/SMAD2/3 signaling pathway [27]. Additionally, exosomes derived from M2-type Mφ carry miR-690, promoting osteogenic differentiation by inhibiting the IRS-1/TAZ signaling pathway [28].

#### 3.1.2. Mesenchymal Stromal Cells

Mesenchymal stromal cells are adult multipotent stem cells characterized by sustained self-renewal and multipotent differentiation capabilities. As one of the most important sources of EVs, mesenchymal stromal cells have significant potential in bone tissue regeneration and anti-inflammatory therapy [29]. Notably, mesenchymal stromal cells can differentiate into various cell lineages, including osteoblasts, chondrocytes, neurons, myogenic cells, and adipocytes [30,31]. Mesenchymal stromal cells, a heterogeneous subgroup of pluripotent stem cells, can be easily isolated from various tissues, including bone marrow, umbilical cord, muscle, and blood [32]. Mesenchymal stromal cells regulate angiogenesis [33], apoptosis [34], proliferation [35], differentiation [36], immune response [37], and microenvironment formation for injury repair [38] by releasing EVs carrying biomolecules [39]. Wang et al. found that mesenchymal stromal cell-derived EVs enhanced BMP/Smad1/5/8 phosphorylation by altering their miRNA expression, which in turn upregulated osteogenic factors such as RUNX2 and ultimately promoted osteogenic differentiation in mesenchymal stromal cells [40]. In vitro, BMSC-derived EVs improve osteogenic function by regulating osteogenic gene expression and osteoblast differentiation. EVs released by human BMSCs can enter osteoblasts and transfer osteogenic miRNAs through endocytosis, thus regulating osteogenic gene expression and promoting osteogenic differentiation. BMSC-derived EVs enhance osteogenic differentiation and gene expression through miR-196a, increasing bone regeneration in cranial defect Sprague Dawley (SD) rats [41]. Wang et al. found that exosome-derived miR-221 and miR-144 significantly decreased during the late stages of bone repair, promoting bone regeneration by enhancing intercellular adhesion molecule-1 expression [42]. EVs can synergize with mesenchymal stromal cells to promote bone regeneration by facilitating relevant biological activities during bone regeneration.

#### 3.1.3. Osteoblasts

Osteoblasts, as one of the most crucial cell sources for promoting bone regeneration, are vital in bone growth and maintenance [43,44], with their biological functions playing key roles in bone formation and repair [45,46,47]. Researchers have found that in primary osteoblasts from mice, their EVs and osteogenesis-related genes exhibit similar gene expression profiles, mainly RUNX2, collagen type I alpha 1 chain (COL1A1), alkaline phosphatase (ALP), and Osterix (OSX) [48]. Cui and colleagues observed significant enhancement of osteogenic marker expression, such as RUNX2 and ALP, after co-culturing mouse bone marrow-derived stromal ST2 cells with MC3T3-derived EVs. These processes enhance matrix bone mineralization by promoting the expression of signaling pathways like Wnt, calcium, and TGF-β [49]. These findings suggest that EVs released by osteoblasts enhance osteogenic differentiation, thereby promoting bone regeneration and mineralization.

#### 3.1.4. Osteoclasts

Due to their resorptive capacity, osteoclasts play a critical role in bone regeneration. When fractures or osteolytic inflammatory diseases occur, osteoclasts are responsible for bone resorption during physiological bone remodeling and pathological bone destruction [49]. Exosomes released by mature osteoclasts can bind to the receptor activator of the NF-κB ligand (RANKL) on the surface of osteoblasts, triggering the reverse RANKL signaling pathway, activating key RUNX2, and promoting bone formation. Additionally, exosomes released from osteoclasts have been found to transport osteoclast–osteoblast coupling factors, and the RANK they secrete binds to RANKL on osteoblasts, activating reverse signaling and increasing RUNX2 activity in osteoblasts and bone formation [50]. Osteoclasts not only resorb and degrade damaged bone tissue but also provide space and conditions for the growth of new bone cells, which is crucial for the bone regeneration process.

Interactions among these cells are essential for maintaining the homeostasis of the bone microenvironment. For example, macrophages, osteoblasts, and osteoclasts accelerate bone loss during the pre-inflammatory phase caused by traumatic fractures by disrupting intercellular homeostasis. At this time, related cytokines are recruited to the injury site, guiding progenitor cells to further differentiate into osteoblasts or osteoclasts to promote bone regeneration. Additionally, EVs produced by different cells can interact with other cells, transfer information, and influence each other. This interaction is crucial for intercellular communication and regulation, potentially helping coordinate different cells to maintain homeostasis. However, under different physiological or pathological conditions, EVs may also disrupt this homeostasis in the body.

### 3.2. EVs Promote Angiogenesis

Angiogenesis plays a crucial role in bone defect repair during bone regeneration. Blood vessels not only supply essential nutrients but also transport and distribute paracrine signals that regulate the growth, differentiation, and regeneration of various cell types, including osteoblasts. Early studies have shown that EVs enhance the production of adipose-derived mesenchymal stromal cells through platelet-derived growth factor (PDGF) and regulate their pro-angiogenic and anti-angiogenic factor content, enhancing the pro-angiogenic activity of EVs in vitro and inducing the formation of capillary-like structures in human microvascular endothelial cells (HMECs) [51]. Additionally, studies on ischemic diseases have shown that mesenchymal stromal cell-derived EVs have strong pro-angiogenic activity [52,53]. Zhang et al. found that EVs released by umbilical cord mesenchymal stromal cells promote angiogenesis by increasing the expression of vascular endothelial growth factor (VEGF) and hypoxia-inducible factor-1α (HIF-1α), ultimately accelerating fracture healing by promoting endothelial cell (EC) proliferation and migration [54]. Furthermore, Zhang et al. discovered that human placenta-derived mesenchymal stromal cell-derived EVs combined with chitosan hydrogel biomaterials increase the stability of proteins (such as VEGF, HGF, and TGF-β) and miRNAs in EVs, increasing their retention time in vivo, and showing enhanced angiogenesis and tissue regeneration in a rat hindlimb ischemia model [55]. Additionally, overexpression of HIF-1α in human dental pulp mesenchymal stromal cells promotes EV release, carrying large amounts of HIF-1α and enhancing angiogenesis through the interaction with the Notch signaling ligand Jagged1 [56]. Xie et al. demonstrated that mesenchymal stromal cell-derived EVs combined with decalcified bone matrix form new functional scaffolds, promoting angiogenesis and enhancing bone regeneration capacity after implantation in mice [57]. The collaboration of EVs with growth factors, cytokines, and biomaterials can enhance angiogenic activity and bone regeneration, significantly benefiting bone regeneration therapy.

### 3.3. EVs Promote Cartilage Regeneration

**Figure 2:**
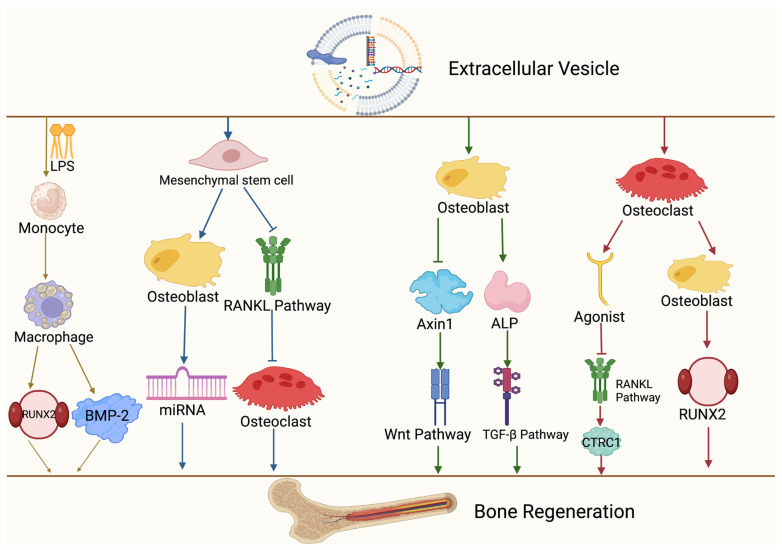
Mechanisms of different cellular sources of EVs in mediating bone regeneration. Extracellular vesicles act on monocytes, mesenchymal stromal cells, osteocytes, and osteoclasts, which in turn activate signaling pathways such as Wnt, TGF-β, and RANKL, and ultimately promote bone regeneration. This figure was created by BioRender (https://app.biorender.com (accessed on 30 July 2024)).

Cartilage formation is essential for bone regeneration. Cartilage promotes fracture healing by filling fracture gaps with cartilage formed by periosteal progenitor cells [58]. During bone regeneration, hypertrophic cartilage is crucial in secondary bone healing with endochondral ossification, as it undergoes mineralization, vascularization, and eventual remodeling into bone after tissue disruption and healing. This process is specific to endochondral ossification and does not apply to intramembranous ossification, where bone formation occurs directly from mesenchymal tissue without the formation of a cartilage intermediate [59]. When EVs from mesenchymal stromal cells were applied to mice with cartilage defects, organized hyaline cartilage filled the cartilage defect surface, promoting cartilage and subchondral bone healing [60]. Chen et al. found that exosomes secreted by chondrocytes have ectopic osteogenic effects in subcutaneous environments, and the subcutaneous injection of EVs in mice efficiently induced ectopic cartilage formation at the structural level, promoting cartilage formation and maintaining cartilage stability [61]. However, cartilage repair has limitations compared to other tissue repairs. Firstly, the number of cartilage tissue cells is low and metabolically inactive. Secondly, cartilage cells have limited capability to obtain nutritional support, relying solely on synovial fluid for nutrient delivery. Thirdly, cartilage is in a hard biomechanical environment affected by tension and friction, hindering the establishment of an effective repair environment [62]. Chondrocyte-derived EVs not only promote osteogenesis by regulating the processes of bone formation and resorption but also facilitate smooth bone regeneration by modulating inflammatory and immune responses. Here, we summarize the mechanisms by which EV-mediated cells impact bone regeneration (Figure 2).

## 4. The Role of EVs in Bone Diseases

### 4.1. Osteoporosis

Osteoporosis (OP) is increasingly recognized as a major global public health issue, particularly as the population ages. The World Health Organization (WHO) has acknowledged osteoporosis as a common disease, and the growing proportion of elderly individuals is expected to exacerbate this concern, leading to higher incidence rates and increased healthcare burdens associated with osteoporosis [63]. OP is a systemic skeletal disease characterized by bone tissue loss, structural damage, and increased bone fragility, making individuals with OP more prone to fractures [64]. OP is primarily associated with the disruption of bone homeostasis. It arises from an imbalance in the processes of bone formation and resorption, leading to a net loss of bone mass. This condition is largely due to the dysregulation of osteoblast and osteoclast activities, which disrupts the dynamic equilibrium necessary for maintaining bone health [65]. Currently, bisphosphonates and denosumab are the first-line clinical treatments to reduce fracture risk, but there is an urgent need for new therapeutic approaches due to their severe side effects and financial burden [66]. Emerging therapies for osteoporosis, such as stem cell transplantation and engineered extracellular vesicle therapy, have garnered significant attention. Pluripotent stem cells such as embryonic stem cells (ESC) and mesenchymal stromal cells have been used in bone regeneration-related fields in a few countries, and it is a very promising direction in the future. Among them, mesenchymal stromal cells are more easily accessible and cost-effective, making clinical application more feasible. However, EVs, which are abundant, easily obtainable, and non-immunogenic, might be more advantageous for clinical application compared to stem cell therapy [67]. Studies have found that in a mouse model of osteoporosis, EVs from BMSCs enhance osteoblast activity by promoting the miR-34c/SATB2 signaling pathway through carrying MALAT1 [68]. Moreover, exosomes from adipose-derived mesenchymal stromal cells reduce bone loss by inhibiting the activation of the NLRP3 inflammasome and the secretion of IL-1β and IL-18 in osteoclasts [69]. The M1-type macrophage-derived exosome miR-98 is associated with increased bone loss in postmenopausal osteoporosis patients. This is thought to occur through the dual effects of the downregulation of DUSP1 and activation of the JNK pathway [70]. Conversely, vascular endothelium-derived exosomes have been shown to reverse the inhibitory effects of glucocorticoids on osteoblasts by inhibiting ferritin autophagy-mediated iron death [71]. In the field of osteoporosis, the efficacy of exosomes derived from different sources varies. Reports have explored the role of exosomes derived from cells, bacteria, plants, and biofluids in OP [72]. The extensive influence of EVs in osteoporosis implies that EVs play a crucial role in the onset, progression, and treatment of OP, providing new strategic options for future treatments.

### 4.2. Osteoarthritis

Osteoarthritis (OA) is a common chronic degenerative joint disease affecting over 300 million people globally, posing a significant health and economic burden. Clinically, it manifests as chronic pain and impaired motor function, severely affecting patients’ quality of life [73]. Primary OA is caused by multiple risk factors, with aging and obesity being the main etiologies. From a pathophysiological perspective, OA is a multifactorial disease involving local cartilage and the entire joint. Current clinical drug treatments primarily aim to alleviate symptoms, with no regulatory-approved drugs available to alter the disease progression of OA [74]. Additionally, OA is a common age-related orthopedic disease similar to osteoporosis, with shared mechanisms and pathological features. Interestingly, subchondral bone loss, characteristic of OP, is also present in the early stages of OA, suggesting that inhibiting OA progression could be approached similarly to OP treatment [75,76]. BMSC-derived exosomes inhibit OA progression by preventing apoptosis of OA chondrocytes [77]. Zheng et al. found that exosomes from normal primary chondrocytes restore mitochondrial function, polarizing macrophage responses to the M2 phenotype. Intra-articular injection of these exosomes effectively inhibited OA onset and progression [78]. Furthermore, TGFβ3-pretreated mesenchymal stromal cell-derived EVs overexpressing miR-455 could promote OA alleviation and cartilage regeneration by activating the SOX11/FOXO signaling pathway [79]. This indicates that EV-mediated OA treatment strategies have great potential, similar to OP treatment. Stem cell-derived EVs regulate the immune response in OA joints through their significant immunomodulatory properties [80]. These EVs reduce inflammation-induced damage by inhibiting the activation of immune cells such as macrophages and the release of pro-inflammatory factors, creating a more favorable environment for tissue regeneration and repair [81].

### 4.3. Fractures

Fractures are one of the most common issues, affecting 2% of the global population, with obesity and low physical activity considered high-risk factors [82]. For fracture patients, bone tissue regeneration is crucial. With increasing understanding of EVs, the roles of EVs from various sources in fractures are being revealed. Hu et al. found that BMSC-derived EVs carrying miR-335 promote osteoblast differentiation and fracture healing by targeting VapB and activating the Wnt/β-catenin pathway [83]. Additionally, BMSC-derived exosomes carrying lncTUG1 promote fracture repair by enhancing osteoblast activity through the miR-22-5p/Anxa8 axis [84]. BMMSC-derived exosomes accelerate endothelial and osteoblast proliferation and migration by activating the HIF-1α/VEGF and BMP-2/Smad1/RUNX2 signaling pathways, promoting angiogenesis and osteogenic differentiation, thus enhancing fracture healing [85]. HIF-1α activation in human umbilical cord mesenchymal stromal cells produces exosomes rich in miR-126, which promote endothelial cell proliferation, migration, and angiogenesis by inhibiting Sprouty-related EVH1 protein activity and activating the Ras/ERK signaling pathway, accelerating fracture healing [86]. EVs promote osteoblast activation and proliferation by carrying growth factors and activating osteogenic pathways, accelerating new bone tissue regeneration and fracture healing. Here, we summarize the roles of EVs in various bone-related diseases (Table 1).

## 5. Clinical Applications of Engineered EVs in Bone Regeneration-Related Diseases

### 5.1. Clinical Diagnostic Tools

Currently, clinical diagnostic tools for bone regeneration and related diseases include X-rays, CT scans, MRIs, and bone density tests. These tools help physicians diagnose bone fractures, osteoporosis (OP), osteoarthritis (OA), and other bone diseases, guiding treatment plans and monitoring treatment efficacy. However, due to the high cost of these tests, radiation exposure, and unreliable conversion markers, better screening methods are essential. With the advancement of EV research, the role of EVs in diagnosing bone regeneration-related diseases is increasingly understood. Osteoporosis: Carriers within EVs, such as proteins, miRNAs, circRNAs, and tRNAs, are often identified as biomarkers for osteoporosis. For example, miRNAs are abundant in many types of cells and enter extracellular fluids via EVs to prevent degradation. Over the past decade, EV-derived miRNAs have become potential biomarkers for the early diagnosis and fracture risk prediction in osteoporosis [87]. Elevated levels of miR-214 in serum EVs of osteoporosis patients suggest that miR-214 in EVs could be a potential biomarker for bone loss [88]. Fractures: Xiong et al. discovered that miR-193a-3p plays a negative role in fracture healing, indicating that miR-193a-3p could be a potential biomarker for predicting non-union fractures [89]. Osteoarthritis: An analysis of plasma and knee joint synovial fluid from early-, middle-, and late-stage OA patients revealed that synovial fluid-derived exosome lncRNA PCGEM1 could differentiate OA progression stages [90]. Additionally, Ali et al. found that circulating miR-320 could distinguish between rapidly progressing, slowly progressing, and non-progressing knee OA, suggesting miR-320 as a potential diagnostic marker for OA [91]. Bone tumors: In chondrosarcoma, researchers identified circulating miR-145 as a potential early diagnostic biomarker for chondrosarcoma subtypes by analyzing the plasma miRNA of patients [92]. Raimondo et al. found that EVs from multiple myeloma (MM) patients deliver increased levels of miR-129-5p to human mesenchymal stromal cells, downregulating ALP and transcription factor Sp1, both positive regulators of osteoblast differentiation, resulting in reduced osteogenic differentiation ability [93]. This suggests that miR-129-5p in EVs could be a potential marker for MM diagnosis and disease progression. miR-501-3p from osteosarcoma cell-derived exosomes promotes osteoclast differentiation by inhibiting PTEN expression and activating the PI3K/Akt signaling pathway, exacerbating bone loss, providing a new target for the clinical diagnosis and treatment of osteosarcoma [94]. Given the limitations of current treatments for bone regeneration-related diseases, discovering more powerful diagnostic biomarkers is crucial for the early diagnosis of these conditions.

### 5.2. Engineered EVs for Treatment

Natural EVs exhibit good biocompatibility, long circulation time in vivo, and low immunorejection. Therefore, engineering them as therapeutic drug carriers in the field of bone regeneration is of significant importance [95,96]. EVs can be used directly for treating bone regeneration-related diseases by carrying biomolecules. Mesenchymal stromal cell-derived EVs promote VEGFA and VEGFR2 expression, facilitating angiogenesis and osteogenic differentiation around bone injuries [97,98]. BMSC-derived EVs influence osteoclast differentiation, alter Mφ polarization states, and TGF-β1 expression, specifically regulating inflammatory immune responses through the OPG–RANKL–RANK signaling pathway, inhibiting periodontitis development and immune damage to periodontal tissues, ultimately promoting periodontal tissue regeneration [99]. Song et al. demonstrated that endothelial cell (EC)-derived EVs have better bone-targeting properties than those derived from osteoblasts or BMSCs, inhibiting osteoclast activity and differentiation via miR-155, suggesting miR-155 as a potential therapeutic target for osteoporosis [100]. Additionally, Hao et al. found that the oral administration of milk-derived EVs inhibited excessive osteoclast differentiation and improved bone mass loss in OVX-induced osteoporotic mice by reducing osteoclastogenesis factors [101]. In osteomyelitis patients and S. aureus-infected MC3T3-E1 mouse clonal osteoblasts, miR-24 expression was downregulated. Conversely, overexpression of miR-24 counteracted the negative effects of S. aureus infection on MC3T3-E1 cells, increasing cell proliferation, reducing apoptosis, and affecting bone formation and differentiation, suggesting that miR-24 could be a potential therapeutic agent for osteomyelitis [102]. However, EVs as therapeutic agents have some limitations, such as weak bone tissue targeting and difficulty in purifying from various vesicle types, which need to be addressed [103].

EVs as drug carriers: EVs can serve as drug carriers for treating bone regeneration-related diseases by carrying small molecules, growth factors, and anti-inflammatory factors. These compounds can be introduced into EVs via co-culture without affecting EV structure, although the nature of the compounds must be considered to influence loading efficiency [104]. Biomaterial-assisted EVs have been reported as therapeutic carriers for bone regeneration, with EV-loaded biomaterial scaffolds overcoming the drawbacks of natural EVs by extending EV storage time and altering release characteristics, improving therapeutic drug acceptance [105]. Qi et al. found that mesenchymal stromal cell-derived exosomes combined with β-tricalcium phosphate (β-TCP) scaffolds promoted mRNA expression of osteogenesis-related genes, enhanced angiogenesis, and osteogenic differentiation in osteoporotic rats, ultimately improving bone regeneration efficiency [106]. BMP2 and macrophage-derived exosome integration with titanium nanotubes activated autophagy, increasing BMP2 expression and creating a favorable environment for mesenchymal stromal cell osteogenic differentiation, promoting bone regeneration [107]. Hao et al. integrated a hydrogel loaded with strontium (Sr), serum exosomes (sEXO), and 3D-printed titanium scaffolds to form a composite material implanted in rabbits, which repaired large bone defects by inhibiting osteoclasts, promoting angiogenesis, and enhancing bone regeneration [108].

Additionally, besides loading exogenous carriers into EVs, specific modifications can be made to EV membranes to enhance targeting and delivery capabilities, overcoming the limitations of EVs as a therapeutic approach. Researchers found that positively charged EVs modified with polylysine–polyethylene–distearoylphosphatidylethanolamine (PPD) can overcome the electrostatic barrier posed by negatively charged cartilage matrixes, making drugs more easily absorbed by chondrocytes, reducing cartilage degradation and wear, and increasing bioavailability [109]. Below, we summarize clinical trials of EVs related to bone regeneration (Table 2).

## 6. Conclusions

As crucial mediators of intercellular communication, extracellular vesicles (EVs) carry various bioactive molecules that regulate the differentiation and activity of osteoblasts, osteoclasts, mesenchymal cells, and immune cells, thereby promoting the growth and repair of bone tissue. Clinical studies have shown that EVs possess significant potential in bone regeneration, becoming a focal point in bone tissue engineering. However, this field also faces numerous challenges. On one hand, the preparation and engineering processes of EVs need to be standardized and optimized to ensure consistent yield, purity, and activity. On the other hand, the stability and targeting of EVs to bone tissue in vivo require thorough investigation. Moreover, EVs might be exploited and lead to pathological processes, such as triggering tumor metastasis and the spread of inflammation [110]. As a clinical therapeutic approach, the development and optimization of EVs are iterative processes. Their bioactivity, pharmacokinetic properties, safety, synthetic feasibility, and dose-dependence in applications are long-term challenges that require extensive clinical trials. However, we believe that with further research into EVs in bone regeneration, improvements through high-throughput screening, nanotechnology, and gene editing will enhance the targeting ability and therapeutic efficacy of engineered EVs. This will result in better targeting, bioactivity, in vivo stability, and feasibility in treatments. Additionally, interdisciplinary collaboration will accelerate the translation of EVs in bone regeneration, addressing the challenges posed by bone tissue damage and diseases more effectively. Through these efforts, EVs are expected to become a significant breakthrough in the field of bone regeneration, providing patients with more effective, safer, and more efficient treatment options, significantly improving their quality of life.

### Materials and Methods

An initial literature search was conducted on 21 February 2024 at Chongqing University School of Medicine. Prior to submission of the manuscript, we re-ran the literature search (16 August 2024) to determine if any new potential articles were included. The following databases were used for the literature search: PubMed, Web of Science, and Scopus. Our search terms were “extracellular vesicles AND bone regeneration”, “exosomes AND bone regeneration”, “extracellular vesicles AND biogenesis”, (extracellular vesicles) AND (osteoporosis OR osteoarthritis OR fracture). The numbers after searching were 7,145,532,238 and 944. Review articles read were mainly published in the last five years, while experimental articles did not clearly differentiate the time. 

## Figures and Tables

**Table 1:** Role of different sources of EVs in bone regeneration-related diseases.

Bone Disease	Role and Specific Effects of EVs	Mechanisms and Pathways	Ref.
Osteoporosis	EVs from MSCs enhance osteoblast activity in osteoporotic mice.	miR-34c/SATB2 axis in BMSC-derived exosomes	[65]
	Adipose MSC-derived exosomes attenuate bone loss by inhibiting osteoclast activity.	Inhibition of NLRP3 inflammasome and reduction of IL-1β and IL-18 secretion	[66]
	M1 macrophage-derived EVs exacerbate bone loss by altering signaling pathways.	Downregulation of DUSP1 and activation of JNK signaling in osteoclasts	[67]
	Vascular endothelial cell-derived exosomes reverse glucocorticoid-induced osteoporosis.	Inhibition of ferritin autophagy-dependent iron concentration	[68]
Osteoarthritis	BMSC-derived exosomes prevent apoptosis and enhance survival of chondrocytes in osteoarthritic environments.	Direct effect on chondrocyte viability,	[74]
	Exosomes from normal primary chondrocytes restore mitochondrial function and influence macrophage behavior.	Polarization of macrophages to M2 phenotype, enhancing repair processes,	[75]
	TGFβ3-pretreated MSC-derived EVs promote remission of OA and regenerate cartilage by modulating cellular pathways.	Activation of SOX11/FOXO signaling pathway,	[76]
	Stem cell-derived EVs modulate immune responses, reducing inflammatory burden in osteoarthritic joints.	Immunomodulatory effects, suppression of pro-inflammatory cytokines	[77,78]

**Table 2:** Clinical trials of EVs in bone regeneration-related diseases.

NCT Number	Title	Status	Conditions	Interventions	Characteristics
NCT06368154	Exosome microRNAs as Potential Biomarkers of Metabolic Bone Disease of Prematurity	Recruiting	• Exosomes • Newborn • Bone diseases, metabolic	Not applicable	Phase: not applicable
NCT05520125	Treatment of Patients with Bone Tissue Defects Using Mesenchymal Stem Cells Enriched by Extracellular Vesicles	Not yet recruiting	• Segmental fracture-bone loss	• Biological: mesenchymal stem cells enriched by extracellular vesicles • Other: standard treatment of bone defects	Phase: • Phase 1 • Phase 2
NCT04998058	Autogenous Mesenchymal Stem Cell Culture-Derived Signalling Molecules as Enhancers of Bone Formation in Bone Grafting	Not yet recruiting	• Bone loss, osteoclastic • Bone loss, alveolar • Alveolar bone loss • Alveolar bone atrophy • Grafting bone	• Procedure: maxillary sinus floor elevation grafting with synthetic bone substitute.	Phase: • Phase 1 • Phase 2
NCT04849429	Intra-discal Injection of Platelet-rich Plasma (PRP) Enriched with Exosomes in Chronic Low Back Pain	Completed	• Chronic low back pain • Degenerative disc disease	• Biological: platelet-rich plasma (PRP) with exosomes • Drug: normal saline	Phase: Phase 1
NCT04281901	Efficacy of Platelet- and Extracellular Vesicle-rich Plasma in Chronic Postsurgical Temporal Bone Inflammations	Completed	• Otitis media chronic • Temporal bone	• Drug: platelet- and extracellular vesicle-rich plasma • Drug: standard conservative treatment	Phase: not applicable
NCT03895216	Identification and Characterization of Predictive Factors of Onset of Bone Metastases in Cancer Patients	Completed	• Bone metastases	Not applicable	Phase: not applicable
NCT03108677	Circulating Exosome RNA in Lung Metastases of Primary High-Grade Osteosarcoma	Active, not recruiting	• Lung metastases • Osteosarcoma	• Other: blood samples	Phase: not applicable

## Data Availability

Not applicable.
